# JETS policy on plagiarism and academic dishonesty

**DOI:** 10.4103/0974-2700.76818

**Published:** 2011

**Authors:** Veronica Tucci, Sagar Galwankar

**Affiliations:** College of Medicine, University of South Florida, Tampa, FL 33620-9951, USA

A journal’s reputation is predicated on its ability to publish high-quality scientific works. Medical journals seek to advance the state of medical art by publishing the highest quality scientific research. Such quality cannot be achieved if plagiarism is abided or if the concept of plagiarism is not fully understood by clinicians, researchers, policy makers, public health workers, and physician-scientists. These scientific manuscripts are laboriously vetted by the peer-review process in order to maintain the public’s trust in our profession and the trust of our readership, authors/contributors, researchers, and reviewers. Unfortunately, the peer-review process does not always have the resources to uncover acts of plagiarism and academic dishonesty and misconduct.

One 2001 study by Rennie and Crosby assessed medical students’ attitudes with respect to matters of academic dishonesty by giving 676 students a questionnaire depicting a fictitious student engaging in 14 separate instances of academic dishonesty.[1] Although the authors note that the surveyed students recognized most of the scenarios to be wrong, of the 461 students who completed the survey, 56% had engaged in or would engage in “copying directly from published text and only listing it as a reference.”[1] Does this signal a disturbing rise of plagiarism and a decline of ethical standards among medical students? It is uncertain whether this trend is the result of the ease at which information including other people’s thoughts and prose can now be pilfered from the Internet or whether there is simply a lack of understanding as to what constitutes plagiarism and the need for clearer guidelines and more express instruction. Nor are medical students the only ones grappling with issues of plagiarism. Scholars, leaders, and even world famous writers are not immune from accusations of plagiarism.[2–5]

We, therefore, feel obliged to establish a policy on plagiarism for the *Journal of Emergencies, Trauma and Shock* (JETS). We also believe it is our obligation to elucidate what is meant by plagiarism so we may help future generations of physician-scientists in their quest to maintain the highest level of personal and professional integrity.

## PLAGIARISM

The Office of Research Integrity (ORI) for the United States Department of Health and Human Services defines plagiarism as “both the theft or misappropriation of intellectual property and the substantial unattributed textual copying of another’s work.”[6] The ORI does not extend its definition of plagiarism to include the “limited use of identical or nearly identical phrases which describe a commonly-used methodology or previous research because ORI does not consider such use as substantially misleading to the reader or of great significance”.[6] Unfortunately, there is no true consensus in the academic community as to what the umbrella of plagiarism includes and the ORI also notes in its brief discussion that ownership of intellectual property is often nebulous.

For our purposes, JETS defines plagiarism as the misappropriation and/or misattribution of ideas and the expression of those ideas, in part or in whole, whether through words, sound, visual displays including photographs, and whether those ideas or expression thereof is taken from published or unpublished abstracts, grants or grant applications, lectures, presentations, Institutional Review Board applications, manuscripts, and articles or excerpts from books or any other published materials or intellectual property.

The editors of JETS appreciate the controversy within the academic community with regard to paraphrasing and the use of nearly identical phrases to explain common methodologies or historical/scientific facts. We, therefore, urge our authors to err on the side of caution and provide citations whenever possible to avoid even the appearance of impropriety and to maintain the highest standard of ethics.

Plagiarism can be subdivided into two categories: (1) one where the author intends to mislead the reader as to author’s contribution by passing off another’s work product as his own; and (2) one where the author does not intend to mislead the reader but misunderstands proper citation or attribution. This second category may be more accurately described as sloppy research work product or “innocent” plagiarism.

The first category includes things such as copying another author’s prose without placing the prose in quotations or offsetting the prose or using italics so as to identify it as the work of another. Both the general public and academic community alike appreciate the sinister nature of such instances of plagiarism. The first category also includes failure to cite the original source even if the author uses quotations or offsets the text. This category also includes using pictures of gross pathology, histopathology, imaging modalities etc. without appropriate attribution if it was not performed by the author’s home institution or affiliated institution. Moreover, even when performed by the home institution, if the author did not personally take the picture, prepare the slide or obtain a copy of the radiograph, the author should include a notation from whom the image was obtained and that it is being used with permission. Although outside the scope of this article, any images that can potentially identify a patient should not be used unless the patient’s express written permission is first obtained and identifying features are removed to the best of the author’s ability.

Unfortunately, items falling into the second category are not as obvious to many researchers and many physicians may be unknowingly plagiarizing in their manuscripts. For example, in providing evidence for a point in his article, a researcher may use an incorrect, imprecise or insufficient citation. This citation may appear because the researcher did not use a software program for keeping track of his footnotes/endnotes and the order was altered accidentally in the final editing process. Although such an alteration would affect all citations downstream of the error, the impact of the “error” is dependent on where in the manuscript it occurred and how many references are included in the manuscript. Anyone who has written a review or original article that has 50, 100, or more references can understand how easily this may occur. It may also be incomplete such as providing a link to a website without noting the date when the information was last accessed as the content and style of information presented online can (and frequently does) change.

In addition, citing a source based on its citation in a later work for support of an author’s premise is also academically dishonest. Before citing any work, the author should review that work in its entirety to ensure that the work indeed supports the author’s contention. Unfortunately, it is not uncommon to see citations of earlier works improperly cited in multiple papers when a later author is using an earlier author’s paper for support and also uses some of the references cited in that earlier author’s paper [Table 1].

**Table 1:** Examples of incorrectly cited text/images

Type of plagiarism	Incorrectly cited text/reference	Correctly cited text/reference
Blatant copying	In the hands of the novice physician, the Fastrach^TM^ may be the most reliable alternate airway device for orotracheal intubation of obese patients.	“[I] n the hands of the novice physician, the Fastrach^TM^ may be the most reliable alternate airway device for orotracheal intubation of obese patients”^1^
	No citation or reference.	1Aikins NL, Ganesh R, Springmann KE, Lunn JJ, Solis-Keus J. Difficult airway management and the novice physician. J Emerg Trauma Shock [serial online] 2010;3:912. Available from: http://www.onlinejets.org/text.asp?2010/3/1/9/58668 [last cited on 2010 Mar 12]
Blatant copying	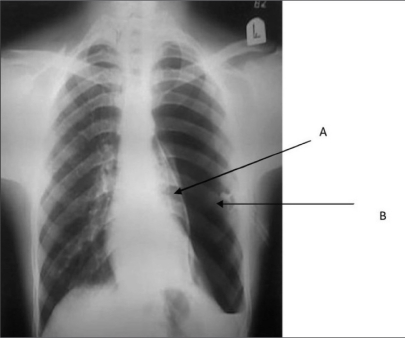	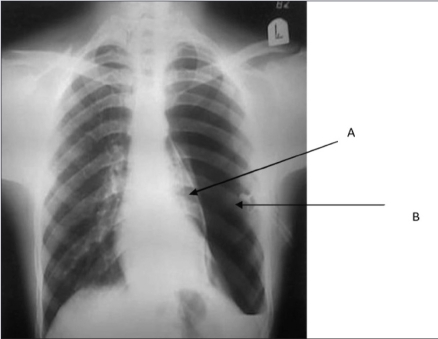
	Pneumothorax secondary to blocked chest tube	Pneumothorax secondary to blocked chest tube.^1^
		1Reprinted with permission from the Journal of Emergency Trauma and Shock, originally published by Sharma A, Jindal P. Principles of diagnosis and management of traumatic pneumothorax. J Emerg Trauma Shock 2008;1:34–41.
Incorrectly citing another text	Although a potentially valuable educational adjunct during clinical training, one study demonstrated that only 58% of medical students felt confident in an examination setting as compared to 88% feeling confident using simulators as a general educational resource.^1^	Although a potentially valuable educational adjunct during clinical training, one study demonstrated that only 58% of medical students felt confident in an examination setting as compared to 88% feeling confident using simulators as a general educational resource.^1^
	1Boulet JR, Murray D, Kras J, Woodhouse J, McAllister J, Ziv A. Reliability and validity of a simulation-based acute care skills assessment for medical students and residents. Anesthesiology 2003;99:1270–80	1Peckler B, Schocken D, Paula R. Simulation in a high stakes clinical performance exam. J Emerg Trauma Shock 2009;2:85–8

Whenever possible an author should not rely on the information provided in a published abstract to cite the article as a whole for support. If the article is not accessible to the author (whether he is unable to obtain a physical copy or if only the abstract is provided in English or a language that the author is able to understand), it is acceptable to provide a citation, noting that the author refers to the abstract and that he was unable to access the paper.

We humbly call on our colleagues across the globe to help us identify and root out any instances of dishonesty or other questionable research practices that have appeared or may in the future, despite our best efforts, appear in the pages of JETS.

The abovementioned examples are not intended to be all-inclusive list and are only provided to serve as a general guide to authors. Authors may also seek examples and further guidance at www.plagiarism.org[7] and www.plagiary.org.[8]

## GUIDELINES ON PLAGARISM AND ACADEMIC DISHONESTY FOR THE JOURNAL OF EMERGENCIES, TRAUMA AND SHOCK

1. Beginning April 1, 2010, JETS will implement this policy for dealing with acts of plagiarism and academic dishonesty.
2. Authors, readers, reviewers and editors are invited to submit accusations of plagiarism in writing along with any supporting documentation or reference to same to editor@onlinejets.org.
3. Upon receipt of an accusation, a committee made up of members of the executive board shall evaluate the accusation including any supporting documentation provided to it within ten (10) business days. During all committee proceedings, editors shall ensure the confidentiality of both the author and the individual(s) who submitted the complaint alleging plagiarism
4. If the committee determines that the evidence is sufficient to warrant a fuller investigation, the committee shall notify the author accused of plagiarizing within ten (10) business days and will give the author a reasonable amount of time to respond to the allegations and procure evidence to support a claim of innocence if necessary.
5. The committee shall review the author’s response, if any, within a reasonable time frame and may elect one of the following actions depending on the nature (ranging from innocent misunderstanding to egregious violation) of the offense:
  - The executive board of JETS acknowledges that there are grey areas of plagiarism such as the ones listed above where the author’s intent is not to mislead. If the author appears to have engaged in merely sloppy research/writing and this is the author’s first offense, the executive board will provide the author with the opportunity to revise his manuscript so that it is in accordance with the highest level of integrity and professionalism. To this end, the executive board will provide the author will guidance on the proper citation/attribution of reference material.
  - If the author submits a second shoddy manuscript after his reeducation or if the committee learns that the author has been previously warned by another journal, a letter of reprimand will be sent to the author with a detailed warning of the consequences for any future incidents and the article may either be revised or withdrawn at the Committee’s discretion.
  - In such cases of substantial plagiarism or academic dishonesty, the committee may deem it appropriate to send a formal letter referring its concerns to the author’s educational institution, professional society, public agency, and/or funding body, with all the commentary and evidence collected by the journal. This will occur when it is believed that genuine misconduct is likely to have occurred (i.e., verbatim wholesale copying of another’s original work).
  - The committee may elect to publish a notice in the journal of redundant or duplicate publication or plagiarism, if appropriate (and unequivocally documented). If the committee elects to alert the JETS readership of an author’s academic dishonesty by publishing a written notice or statement, it may do so without will not providing advance notice or obtaining permission from the author in question.
  - In addition to any of the sanctions noted above, a plagiarized manuscript will be formally withdrawn, removed or otherwise retracted from the journal’s electronic archives and from the scientific literature including the indexing authorities (PubMed, MEDLINE, etc) to the extent that the committee is able.

It was unanimously decided that the editors have the responsibility to promote the highest level of ethics and academic integrity in the field of emergency medicine. We have developed this policy on plagiarism to urge our fellow researchers and physician-scientists to properly cite our colleagues’ work product, thereby, recognizing the effort put into developing such research and the contribution that research makes to our field of medicine.

Any inquiries regarding this policy or future updates should be directed to editor@onlinejets.org
